# Pioglitazone-induced AMPK-Glutaminase-1 prevents high glucose-induced pancreatic β-cell dysfunction by glutathione antioxidant system

**DOI:** 10.1016/j.redox.2021.102029

**Published:** 2021-06-03

**Authors:** Udayakumar Karunakaran, Suma Elumalai, Jun Sung Moon, Kyu Chang Won

**Affiliations:** Department of Internal Medicine, Yeungnam University College of Medicine, Daegu, Republic of Korea

**Keywords:** Pioglitazone, AMPK, Endoplasmic reticulum stress, Oxidative stress, Glutaminase, Diabetes

## Abstract

Prolonged hyperglycemia plays a major role in the progression of β-cell loss in diabetes mellitus. Here we report an insulin sensitizer thiazolidinedione Pioglitazone selectively preserves the beta cells against high glucose-induced dysfunction by activation of AMPK and Glutaminase 1 (GLS1) axis. AMPK activation increases the stability of Glutaminase 1 by HSP90 family mitochondrial heat shock protein 75 (HSP75/TRAP1). This is associated with an elevation of GSH/GSSG ratio which leads to inhibition of mitochondrial dysfunction by induction of BCL2/BCL-XL in high glucose conditions. Pioglitazone was able to also protect against high glucose-induced elevations in maladaptive ER stress markers and increase the adaptive unfolded protein response (UPR) by inhibiting mTORC1-eEF2 protein translation machinery. Moreover, the pioglitazone effect on AMPK activation was not dependent on the PPARγ pathway. Strikingly, chemical inhibition of AMPK signaling or glutaminase-1 inhibition abrogates the pioglitazone effect on the TRAP1-GLS1 axis and GSH/GSSG ratio linked to mitochondrial dysfunction. Finally, inhibition of AMPK signaling enhanced maladaptive ER stress markers by mTORC1-eEF2 activation. Altogether, these results support the proposal that pioglitazone induced AMPK activation stabilizes a novel interaction of TRAP1/HSP75-GLS1 and its downstream signaling leads to improved β-cell function and survival under high glucose conditions.

## Highlights

• Pioglitazone activates AMPK independent of PPARγ.
• AMPK activation induces Glutaminase-1 (GLS1) stability by TRAP1/HSP75.
• GLS1 activation enhances GSH antioxidant system.
• AMPK inhibit mTORC1-eEF2 axis and reduce ER stress.

## Graphical abstract

**Figure:**
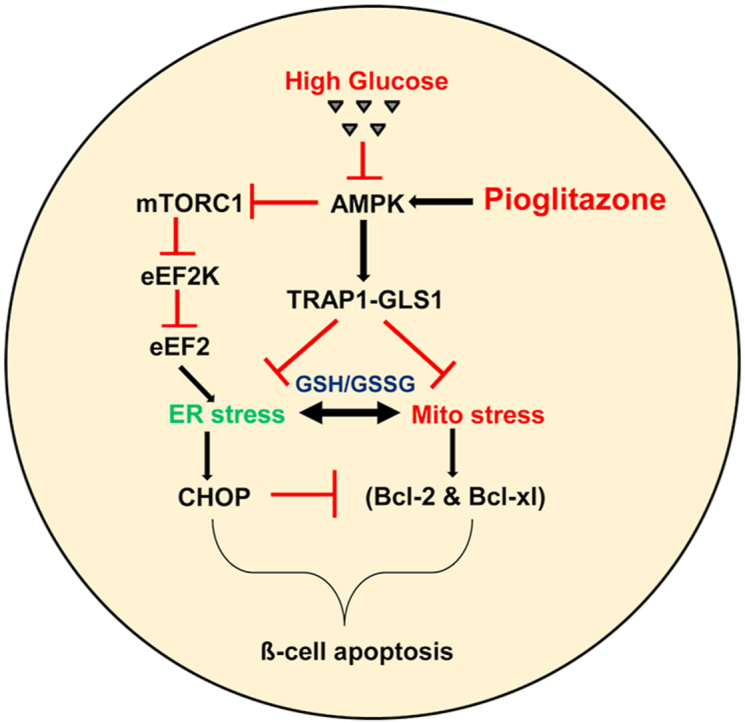

## 1 Introduction

Thiazolidinediones (TZDs), a class of antidiabetic agents that act by improving glucose homeostasis via enhancement of insulin sensitivity and preservation of islet architecture and function in various animal models of obesity and diabetes [[1], [2], [3], [4], [5], [6]]. On the other hand, pioglitazone acutely slows down glucose and lipid metabolism in the beta-cell and inhibits glucose-induced insulin secretion (GIIS) primarily at submaximal and much less at maximal glucose concentrations via a PPARγ-independent mechanism [7]. These acute effects of pioglitazone are likely attributable to complex I inhibition of the electron transport chain [8] or through the inhibition of mitochondrial pyruvate carrier and involve reduced glucose oxidation, decreased ATP levels, and increased AMPK activation [9]. Moreover, pioglitazone exerts a direct action on the beta-cell to reduce insulin secretion most likely by causing mild metabolic restriction/deceleration [10]. In addition, pioglitazone improves beta-cell function and survival in NOD mice by enhancing the unfolded protein response and rescues early glycemic deterioration and beta-cell death [11]. Consistent with this mechanism of action, pioglitazone exert a direct effect in diabetic islets resulted in the reduction of endoplasmic reticulum stress leading to favorable alterations of the islet gene chromatin architecture in db/db mice [12]. To facilitate this process further, it would be important to pinpoint regulatory mechanisms that are relevant under physiological and pathological conditions. In particular, understanding the mechanisms involved in dictating specific pioglitazone downstream cellular outcomes will aid in the discovery of potent target specific downstream signaling cascades.

## 2 Materials & methods

### 2.1 Cell culture

The INS-1 rat insulinoma cell line (INS-1) and Human pancreatic insulin-releasing 1.1B4 cells (passage 20–30; purchased from ECACC, European Collection of Cell Cultures, Sigma-Aldrich, St Louis,MO,USA) were cultured in 5% CO_2_ at 37 °C in RPMI 1640 (Life Technologies, Inc., Grand Island, NY) medium supplemented with 10% fetal bovine serum (FBS) (Gibco, Grand Island, NY, USA), 100 units/ml penicillin, and 100 μg/ml streptomycin. We subjected INS-cells to high glucose (30 mM) for 36hrs with or without pioglitazone (10 μM). Human pancreatic beta cells were exposed to high glucose (30 mM) for 48hrs with or without pioglitazone (10 μM). After 48hrs, the percentage of viable cells were measured using the Cell Counting Kit-8 (CCK-8) (Dojindo Lab., Kumamoto, Japan). GW9662 (PPARγ antagonist) was purchased from Cayman (USA) and AMPK inhibitor (BML-275) from Enzo Life Sciences (USA). MPC inhibitor (UK5099), Glutaminase inhibitor (BPTES), Glutathione synthesis inhibitor (BSO) and Proteasomal inhibitor MG132 were purchased from Sigma Aldrich (USA).

### 2.2 Cell apoptosis, ROS, MMP and GSH/GSSG assay

INS-cells were exposed to high glucose (30 mM) for 36hrs with or without pioglitazone (10 μM). After 36hrs, apoptosis was assessed using the TUNEL In-situ cell death detection kit (Roche, Basel, Switzerland), according to the manufacturer's instructions. Images were captured using a fluorescence microscope. Intracellular ROS production (especially superoxide and hydroxyl radicals) in cells was estimated using the fluorometric intracellular ROS assay kit (MAK144; Sigma) Fluorescence signals were read on a fluorescence microplate reader (Molecular Devices) at the excitation and emission wavelengths of 540 and 570 nm, respectively. The mitochondrial membrane potential was measured using DiOC6 (Sigma-Aldrich). Stained cells were washed twice and resuspended in PBS for analysis by flow cytometry. Total GSH and GSSG levels were measured by Glutathione detection kit according to the manufacturer's instructions (Enzo Life Sciences Inc, Farmingdale, NY, USA).

### 2.3 Western blotting

Cell protein lysates were resolved using NuPAGE 4–12% Bis-Tris gel (Invitrogen) and transferred to PVDF membranes (Millipore, Billerica, MA, USA). After blocking, the membranes were stored at 4 °C with the following primary antibodies: AMPK(1:1000), ACC(1:1000), mTORC1 (1:1000), p70S6K(1:1000), eEF2K(1:1000), eEF2 (1:1000), ATF4 (1:1000), eIF2α(1:1000), CHOP(1:1000), BCL-2 (1:1000), BCL-XL (1:1000), and cleaved caspase 3 (1:1000), (Cell signaling Technology, Danvers, MA, USA), HSP75/TRAP1 (1:1000), cytochrome *c* (1:2000), (BD Biosciences, San Jose, CA, USA), P35 (1:1000), CDK5 (1:1000), (Santa Cruz, Dallas, TX, USA), GLS1 (1:2000), (Proteintech, Rosemont, IL, USA), p66Shc (1:1000), and actin (1:5000), (Abcam, Cambridge, UK). The membranes were then washed and incubated with horseradish peroxidase (HRP)-conjugated secondary antibodies. Immuno-reactive proteins were detected using ECL reagents (ECL Plus; Amersham, GE Healthcare Life Sciences, Little Chalfont, Buckinghamshire, UK). Cell protein lysates were collected, and co-immunoprecipitation was performed using GLS-1 antibody.

### 2.4 Data analysis

Statistical significance was determined with the Student's *t*-test or by analysis of variance (ANOVA) as appropriate. A value of p < 0.05 was indicated statistical significant.

## 3 Results

### 3.1 Pioglitazone effect on AMPK activation

**Fig. 1:**
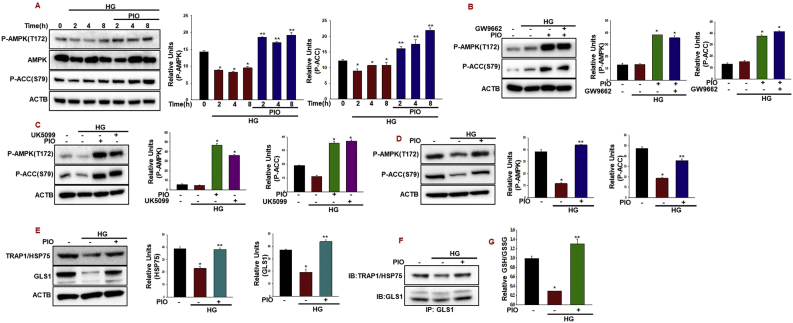
**Pioglitazone effect on AMPK activation. (**A) The INS-1 cells were treated with pioglitazone (10 μM) with or without high glucose (30 mM) for the indicated time periods. Pioglitazone-induced AMPKα (Thr172) and ACC (Ser79) phosphorylation was analyzed with western blotting (*P < 0.05 vs control; **P < 0.05 vs HG). (B) INS-1 cells were incubated in the presence of PPARγ antagonist GW9662 (10 μM) with or without Pioglitazone (10 μM) along with high glucose (30 mM) for 2 h. (*P < 0.005 vs HG). (C) The INS-1 cells were treated with mitochondrial pyruvate carrier inhibitor UK5099 (10 μM) for 1 h and then exposed to high glucose (30 mM) for 2 h (*P < 0.005 vs HG). (D) Immunoblots for Pioglitazone-induced AMPKα (Thr172) and ACC (Ser79) phosphorylation after 36 h. (*P < 0.005 vs control; **P < 0.05 vs HG). (E) INS-1 cells were treated with high glucose (30 mM) with Pioglitazone (10 μM) for 36 h and TRAP1/HSP75 and GLS1 protein levels were quantified by immunoblotting (*P < 0.05 vs control; **P < 0.05 vs HG). (F) Co-IP of GLS1 with TRAP1 after pioglitazone treatment in high glucose conditions. (G) Measurement of relative GSH/GSSG ratios in INS-1 cells after pioglitazone treatment in high glucose conditions after 36 h (*P < 0.001 vs. Control, **P < 0.005 vs. HG).

We assessed the acute effect of pioglitazone on AMPK activation using INS-1 cells. As shown in Fig. 1A, incubation of INS-1 cells to high glucose for different time points resulted in a decrease in AMPK activity as evidenced by the decrease in phosphorylation of its downstream target ACC. Pioglitazone treatment caused an increase in AMPK activity which paralleled the increase in ACC phosphorylation. To assess whether the effects of pioglitazone on AMPK activation is mediated via PPARγ, we used the PPARγ antagonist GW9662. Inhibition of PPARγ using GW9662 does not inhibit the phosphorylation status of both AMPK and ACC by pioglitazone in the early (Fig. 1B) or later time point (Supplemental Figure. 1A) suggesting the PPARγ independent effect of Pioglitazone. To understand whether altering mitochondrial pyruvate carrier modulated AMPK activation by pioglitazone, we treated INS-1 cells with mitochondrial pyruvate carrier inhibitor UK5099 under high glucose conditions. Addition of the mitochondrial pyruvate carrier inhibitor UK5099 showed increased AMPK activity as evidenced by the increase in phosphorylation of its downstream target ACC suggest that the blockade of pyruvate entry into mitochondria results in bioenergetics crisis, possibly because of increases in AMP to ATP ratio in INS-1 cells, leading to AMPK activation (Fig. 1C). Over the acute time period studied here, we then addressed the question whether pioglitazone induced AMPK activity was retained for the 36 h experimental duration with high glucose conditions. Although high glucose caused a significant decrease in AMPK activity, pioglitazone completely restored it (Fig. 1D). Recently several papers have reported that TRAP1, the mitochondrial isoform of the heat shock protein (HSP90), inversely correlated with OXPHOS-coupled ATP synthesis in different normal and cancer cell types [[13], [14], [15], [16]]. Besides this regulation, TRAP1 is also localized in the endoplasmic reticulum (ER) to yield protection from ER stress, oxidative damage and cell death [17,18]. However, the role of TRAP1 in the pancreatic beta cells, remains unknown. Therefore, we first determined the TRAP1 stability after pioglitazone treatment in high glucose conditions. Interestingly, TRAP1 protein stability was decreased in high glucose treated cells and pioglitazone treatment (Fig. 1E) or proteasome specific inhibitor MG132 rescued high glucose induced TRAP1 degradation in INS-1 cells, indicating that ubiquitination induces TRAP1 degradation through a proteasomal pathway (Supplemental Fig. 1B). Moreover, inhibition of mitochondrial pyruvate carrier enable cells to utilize glutamine metabolism (glutaminolysis) to generate mitochondrial intermediates for tumor growth and survival [19]. Exposure of INS-1 cells to high glucose reduced the protein levels of GLS1. Interestingly, Pioglitazone treatment induced the protein levels of GLS1 at high glucose conditions (Fig. 1E). Functionally, CoIP experiments showed that TRAP1 interacted with GLS1. Remarkably, their interaction was decreased under high glucose conditions (Fig. 1F). Moreover, glutamine dependent glutathione production is important for controlling the accumulation of mitochondrial reduced oxygen species (ROS) and also positively correlated with insulin secretion [20]. We subsequently examined whether this interaction impacts the GSH/GSSG ratio in cells after pioglitazone treatment. Importantly, the GSH/GSSG ratio was clearly increased in pioglitazone treated cells compared with high glucose conditions (Fig. 1G).

### 3.2 Pioglitazone inhibit high glucose induced mitochondrial dysfunction

**Fig. 2:**
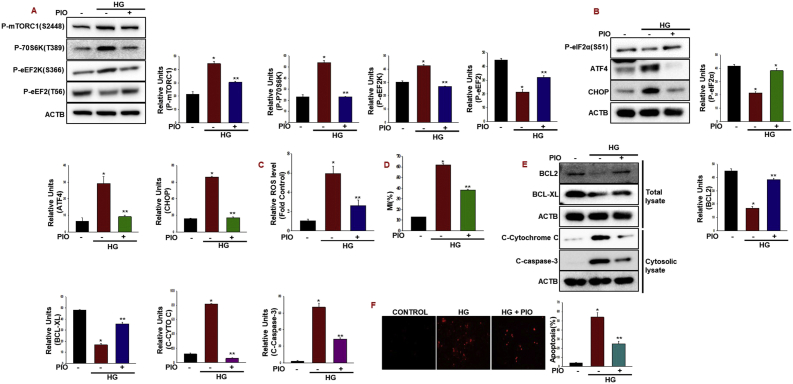
**Pioglitazone inhibit high glucose induced mitochondrial dysfunction.** (A, B) INS-1 cells were treated with high glucose (30 mM) with Pioglitazone (10 μM) for 36 h and protein levels were quantified by immunoblotting (A) (*P < 0.05 vs. Control, **P < 0.05 vs. HG). (B) (*P < 0.01 vs. Control, **P < 0.01 vs. HG).(C, D) Cells were treated as mentioned above and cellular ROS and mitochondrial membrane potential was analyzed by fluorometric intracellular ROS assay and mitochondrial membrane potential was analyzed by using DiO_6_ dye. (*P < 0.001 vs control; **P < 0.001 vs HG). (E) BCL-2, BCL-XL, Cytosolic cytochrome *c* and cleaved casapase-3 protein levels were quantified by immunoblotting. (*P < 0.01 vs. Control, **P < 0.01 vs. HG). (F) INS-1 cells were treated with high glucose (30 mM) with Pioglitazone (10 μM) for 36 h and cell apoptosis was analyzed by TUNEL assay (*P < 0.001 vs control; **P < 0.001 vs HG). All data are expressed as the mean ± SEM of at least three independent experiments.

On the other hand, metabolic response to nutrients is tightly regulated by nutrient sensing mechanisms that inhibition of AMPK in beta cells by high glucose inversely correlates with activation of the mammalian Target of Rapamycin (mTOR) pathway coordinate with enhanced protein translation coupled with cell death [[21], [22], [23]]. In this context, we observed that pioglitazone inhibits the mTOR pathway by regulating the activation of the eukaryotic elongation factor 2 kinase (eEF2K) leads to the phosphorylation of its target eEF2 and inhibit the protein translation elongation (Fig. 2A). Under the same conditions, we observed the decreased maladaptive ER stress markers phospho eIF2α, ATF4, and CHOP expression in pioglitazone treated cells compared to high glucose treated cells (Fig. 2B). As expected, exposure of INS-1 cells to pioglitazone reduced the cellular ROS production, mitochondrial membrane potential loss and cleaved caspase-3 activation and at the same time enhanced the antiapoptotic BCL2, BCL-XL proteins and reduced the cell death by high glucose (Fig. 2C–F). However, it has been well established that BCL-2 and its relative BCL-XL can block most forms of apoptotic cell death by preventing mitochondrial dysfunction [24]. Collectively, these results establish a link between TRAP1-GLS1 in pioglitazone mediated beta cell protection and decreases of mTOR flux during high glucose stress.

### 3.3 AMPK inhibition reverse Pioglitazone protective effect in beta cells

**Fig. 3:**
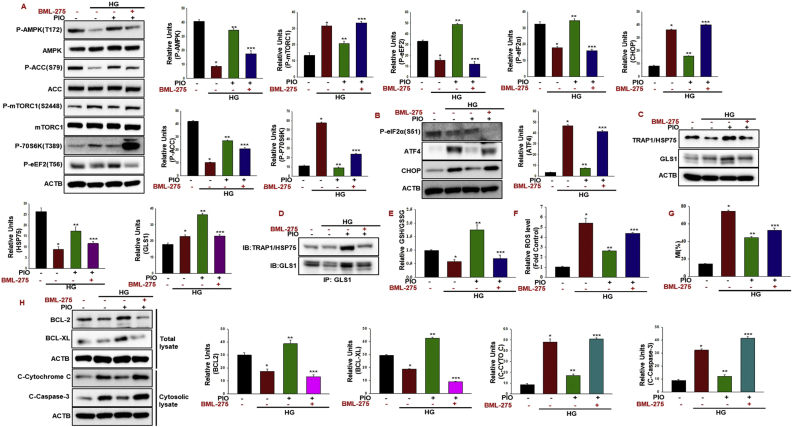
**Effect of pioglitazone in the absence of AMPK on high glucose induced mitochondrial dysfunction.**(A–C) INS-1 cells were treated with high glucose (30 mM) with Pioglitazone (10 μM) for 36 h with or without BML-275 (10 μM). The cell extracts were harvested and tested for protein levels with indicated antibodies. Actin was used as the loading control. (A) (*P < 0.001 vs control; **P < 0.001 vs HG; ***P < 0.01 vs PIO) (B) (*P < 0.001 vs control; **P < 0.001 vs HG; ***P < 0.001 vs PIO) (C) *P < 0.05 vs control; **P < 0.05 vs HG; ***P < 0.05 vs PIO) (D) Co-IP of GLS1 with TRAP1 after pioglitazone treatment with BML-275 (10 μM) in high glucose conditions. (E) Measurement of relative GSH/GSSG ratios in INS-1 cells after pioglitazone treatment with BML-275 (10 μM) in high glucose conditions after 36 h (*P < 0.001 vs. control; **P < 0.005 vs. HG; ***P < 0.005 PIO). (F, G) Cellular ROS production and mitochondrial membrane potential after BML-275 treatment. (H) Cytosolic cytochrome *c*, cleaved casapase-3, BCL-2 and BCL-XL protein levels were quantified by immunoblotting (*P < 0.01 vs control; **P < 0.01 vs HG; ***P < 0.01 vs PIO). Data are represented as mean ± SEM of three independent experiments.

We speculated that the inhibition of AMPK might impair pioglitazone's protective effect against high glucose. To test this possibility, we used BML-275, a potent and selective AMPK inhibitor. As was shown, BML-275 treatment prominently repressed pioglitazone-induced AMPK activity under high glucose conditions paralleled with the activation of mTOR phosphorylation and its downstream target p70S6-eEF2 kinase (Fig. 3A). Consequently, increased mTOR phosphorylation coincide with increased ER stress markers such as phospho eIF2α, ATF4, and CHOP (Fig. 3B). Consistent with what has been observed for ER stress markers, TRAP1-GLS1 protein levels and their interactions were decreased in AMPK inhibited cells hypothesized that AMPK activation might be responsible for the stability of TRAP1-GLS-1 proteins after pioglitazone treatment (Fig. 3C and D). Remarkably, BML-275 treatment reduced the GSH/GSSG ratio in pioglitazone treated cells (Fig. 3E). The importance of AMPK activation by pioglitazone on mitochondrial function was also evident in our studies with cellular ROS production. As shown in Fig. 3F, BML-275 treatment prominently repressed Pioglitazone effect on intracellular ROS production under high glucose conditions. Furthermore, the mitochondrial membrane potential loss was increased after BML-275 treatment with pioglitazone (Fig. 3G). However, it has been well established that BCL-2 and its relative BCL-XL can block most forms of apoptotic cell death by preventing mitochondrial dysfunction [24]. Consistent with this, we found that pioglitazone remarkably inhibited the reduction of BCL-2 and BCL-XL by high glucose and inhibition of AMPK by BML-275 reversed the Pioglitazone effect on BCL-2 and BCL-XL protein levels (Fig. 3H). To a similar extent, inhibition of AMPK with BML-275 significantly increased cleaved caspase-3 activity (Fig. 3H).

### 3.4 Glutaminse and glutathione synthesis inhibition reverse Pioglitazone protective effect in beta cells

**Fig. 4:**
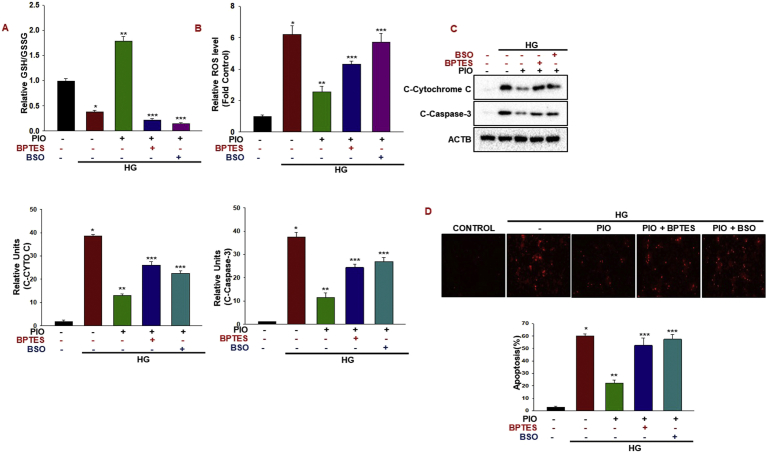
**Glutaminse and glutathione synthesis inhibition reverse Pioglitazone protective effect in beta cells.** (A–D) INS-1 cells were treated with high glucose (30 mM) with Pioglitazone (10 μM) for 36 h with or without Glutaminase 1 inhibitor BPTES (10 μM) or glutathione synthesis inhibitor BSO (100 μM). Measurement of relative GSH/GSSG ratios and cellular ROS production were measured (*P < 0.001 vs. control; **P < 0.005 vs. HG; ***P < 0.005 PIO). Cytosolic cytochrome *c*, cleaved casapase-3, protein levels were quantified by immunoblotting (*P < 0.001 vs control; **P < 0.005 vs HG; ***P < 0.01 vs PIO). (D) Cell apoptosis was analyzed by TUNEL assay (*P < 0.001 vs control; **P < 0.001 vs HG; ***P < 0.001 vs PIO).

To investigate the role of Glutaminse-1 signaling to induce glutathione synthesis in our experimental conditions, we treated the cells with the Glutaminse-1 inhibitor BPTES or GSH synthesis inhibitor buthionine sulfoximine (BSO) with pioglitazone. We observed that the BPTES and GSH synthesis inhibitor synergistically decreased the pioglitazone effect on GSH/GSSG ratio (Fig. 4A). The decreased GSH/GSSG ratio in inhibitor-treated cells suggests that the ROS levels have accumulated to potentially produce an oxidative stress that can hinder the protective effect of pioglitazone. Similar negative results also obtained with the intracellular ROS production after inhibitors treatment with pioglitazone (Fig. 4B). This indicates that a potential reason for failure of pioglitazone to impact the growth of beta cells is due to an increased ROS levels. Accordingly, we observed greatly enhanced caspase-3 activation in BPTES and BSO treated cells (Fig. 4C). We postulated that beta cells would be more sensitive to cell death in pioglitazone treated with BPTES or BSO due to their decreased GSH levels (Fig. 4D). As previously observed for high glucose, BSO or BPTES induced a significant decrease of reduced GSH in pioglitazone treated cells, indicating that glutathione dependent H2O2 scavenging activity was impaired. In addition we found that inhibition of GLS1 enhances pioglitazone effect on AMPK, TRAP1 suggesting that GLS1 acts downstream of AMPK signaling (Supplemental Fig. 2A).

### 3.5 Pioglitazone effect on AMPK-GLS1 activation in human pancreatic beta 1.1b4 cells

**Fig. 5:**
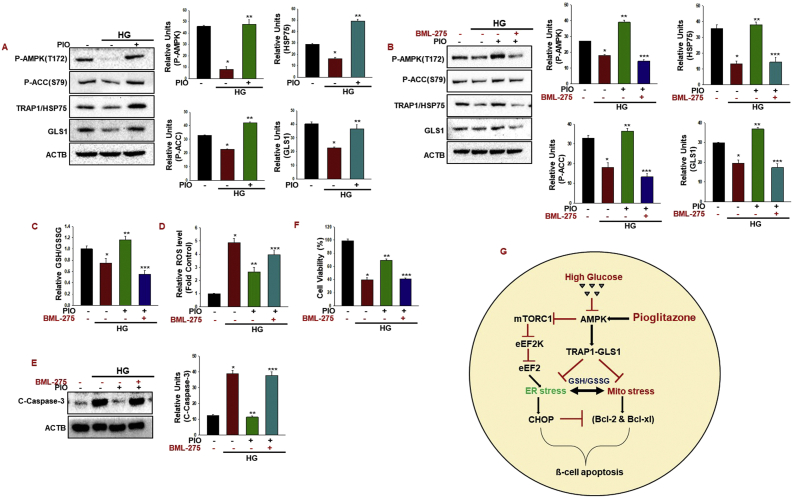
**Pioglitazone effect on AMPK-GLS1 activation in Human pancreatic beta 1.1b4 cells.** (A) Human pancreatic 1.1b4 cells were incubated with Pioglitazone (10 μM) for 48 h with high glucose (30 mM). Pioglitazone-induced AMPKα (Thr172) and ACC (Ser79), TRAP1/HSP75, and GLS1 was analyzed with western blotting (*P < 0.001 vs control; **P < 0.005 vs HG). (B) Human pancreatic 1.1b4 cells were treated with high glucose (30 mM) with Pioglitazone (10 μM) for 48 h with or without BML-275 (10 μM). The cell extracts were harvested and tested for protein levels with indicated antibodies (*P < 0.001 vs control; **P < 0.001 vs HG; ***P < 0.001 vs PIO). (C, D) Measurement of relative GSH/GSSG ratios and cellular ROS production were measured (*P < 0.001 vs. control; **P < 0.005 vs. HG; ***P < 0.005 PIO). (E, F) Cleaved caspase-3 and cell viability were measured using the Cell Counting Kit-8 (*P < 0.01 vs control; **P < 0.005 vs HG; ***P < 0.005 vs PIO). (G) Pioglitazone activates AMPK, which causes a TRAP1/HSP75-GLS1 interaction, which increases the GSH/GSSG ratio and blocks mTORC1-induced maladaptive ER stress, protecting cells from high glucose toxicity.

In an attempt to establish the effects of pioglitazone on AMPK activation in human pancreatic beta cells, we treated 1.1b4 cells with high glucose with or without pioglitazone for 48 h. As expected, treatment with pioglitazone resulted in AMPK activation. Interestingly, TRAP1 and GLS1 protein stability was maintained in pioglitazone-treated cells (Fig. 5A). Combined, these results indicate that AMPK activation blocks TRAP1 and GLS1 degradation. Besides, treatment with BML-275 resulted in marked AMPK inhibition. We further detected that treatment with BML-275 decreased the TRAP1 and GLS1 stability (Fig. 5B). These results indicate that the AMPK activity in 1.1b4 cells was also increased by pioglitazone and that inhibition of AMPK activity reduced the GSH/GSSG ratio with the upregulation ROS production (Fig. 5C–D). Consequently, inhibition of AMPK activity by high glucose increased cleaved caspase-3 expression and induce cell viability loss in the pioglitazone-treated 1.1b4 cells (Fig. 5E–F). Collectively, we describe a mechanism by which pioglitazone responds to high glucose conditions through induction of AMPK, which controls TRAP1-GLS1 activation. Thus, the increased TRAP1-GLS1 axis protects the cells from high glucose-induced mitochondria-ER dysfunction and cell death (Fig. 5G).

## 4 Discussion

In this study, we have shown that pioglitazone induced AMPK activation enhance TRAP1-GLS1 interaction which alleviates the high glucose induced beta cell mitochondrial oxidative damage and apoptosis. Considering the known functions of pioglitazone in orchestrating glucose and lipid metabolism have direct effects to improve pancreatic beta cell function and survival in T2DM [25]. It is conceivable that the antidiabetic effects of thiazolidinedione may be mediated in part through the inhibition of mitochondrial pyruvate carrier and activation of AMPK [9,26,27]. In support of this model, our results showed that pioglitazone increased AMPK activation possibly due to the inhibition of mitochondrial pyruvate carrier. Recently, Nemani et al. [28] showed enhanced AMPK activation in mitochondrial pyruvate carrier knockout cells, leading to cell survival under nutrition stress. Moreover, inhibition of pyruvate carrier enhance the glutamine utilization (Glutaminolysis) which drives cell proliferation and maintains redox balance for cell survival [19,29]. In contrast, glucose inhibit the pathway of glutaminolysis and impaired its capability to induce insulin secretion [30]. Besides, glutamine provides precursors for the GSH biosynthesis pathway critical for the attenuation of ROS which plays a crucial role for cell survival [31]. Thus, our data have also provided evidence that pioglitazone maintains the cellular redox balance by reducing GSSG to GSH to prevent ROS-mediated damage. In particular, glutamine supplementation has been shown to induce the heat shock protein HSP70 and GSH expression and protect the islets against the ischemic damage [32]. It is postulated that TRAP1/HSP75 overexpression inhibits oxidative stress induced Saos-2 osteosarcoma cells death by inducing the levels of reduced glutathione [33]. It has been reported that Glutaminase 1 (GLS1) directly senses glutamine availability under glucose deprivation to maintain mitochondrial integrity [29]. Our results also demonstrate that GLS1 increase upon pioglitazone challenge is an adaptive signaling associated with changes in the GSH production upon glucose stress which involves mitochondrial pyruvate carrier as an intermediate player. Moreover, pioglitazone increase the stability of GLS1 by TRAP1 and thus protect the cells against high glucose by glutathione redox system. To further dissect the mechanism underlying AMPK activation, we assessed the expression and activity of mTORC1 under high glucose conditions. Recent studies demonstrate that constitutive mTORC1 activation enhances the immature phenotype of beta cells. However, activation of AMPK signaling increases the functional maturation of beta cells via improvement of mitochondrial biogenesis [34]. In addition, consequent activation of mTORC1 caused enhanced protein translational ER stress, leading to increased vulnerability of cells to apoptotic cell death [[21], [22], [23]]. Chronic mTORC1 signaling associated with enhanced ER stress-triggered transcriptional induction through ATF4 and CHOP increases protein synthesis leading to oxidative stress and cell death [35]. mTORC1-p70 ribosomal protein S6 kinase (p70S6K) signaling pathways control protein synthesis via eukaryotic elongation factor 2 (eEF2). Pioglitazone-mediated p70S6K inhibition decreases eEF2K phosphorylation and inhibit protein synthesis by eEF2 (T56) phosphorylation leading to the attenuation of stress (Fig. 2D). Inhibition of AMPK activates mTORC1 signaling causes apoptosis through suppression of eEF2K and consequent induction of ER stress by eEF2 (Fig. 3A). Remarkably, pioglitazone treatment reduced the protein translation and recapitulating the adaptation with downregulated ER stress markers. While preparing our manuscript we found an interesting article that inhibition of mitochondrial pyruvate transport using (UK5099) reduces mitochondrial nutrient overload and allows beta cells to recover from chronic glucose stress [36]. These results seem to be relevant to our hypothesis that MPC inhibition activates AMPK which inhibits high glucose effect via TRAP1-GLS1, and raise a possibility that GLS1 induction may suppress beta cell dysfunction via GSH/GSSH ratio. Moreover, it has been known that TRAP1 regulates the rate of protein synthesis through the eIF2α pathway and attenuation of cap-dependent translation under stress through enhances the synthesis of selective stress-responsive proteins, such as BiP/Grp78, and the cystine antiporter system xCT, thereby providing protection against ER stress [37]. The cystine antiporter system xCT, is the rate-limiting step in the glutathione (GSH) biosynthesis pathway that plays a significant role in antioxidative defense [38]. Based on these observations, it can be speculated that AMPK mediated TRAP1-GLS1 interaction increases the cellular GSH/GSSG ratio and inhibition of AMPK increased the degradation of TRAP1 leads to decreased GLS1 and GSH/GSSH ratio. Moreover, Kono et al. [39] used β-cells to demonstrate that a high glucose and the inflammatory cytokine-mediated phosphorylation of PPAR-γ at Ser 273 by CDK5, leads to decreased expression of SERCA2b, a major ER Ca^2+^ pump protein. Our previous study has shown that inhibition of CDK5 in beta cells decreased the levels of mitochondrial oxidative damage and apoptosis induced by high glucose, as indicated by reduced p66Shc phosphorylation and superoxide levels [40,41]. Owing to its link to beta cell pathogenesis, we exposed beta cells to high glucose with pioglitazone and found that pioglitazone inhibit CDK5 tyrosine 15 phosphorylation which is closely associated with the p66Shc activation (Supplemental Fig.3A). In addition, AMPK inhibition reversed the pioglitazone mediated CDK5 inhibition (Supplemental Fig.3B). Our findings complement all these data and provide an explanation why AMPK-GLS1 activation what we believe is a novel mechanism for the regulation of pancreatic beta cell function by pioglitazone related to oxidative stress under high glucose conditions.

## 5 Conclusion

Pioglitazone activates AMPK which inhibits high glucose effect via enhancing the Gluatminase-1 stability by HSP75/TRAP1, and reestablish a possibility that Glutaminase-1 (GLS1) stability may suppress beta cell dysfunction by glutathione antioxidant system. Hence, a broader understanding of AMPK-GLS1 biochemical pathways under metabolic stress needed for therapeutic targeting of beta cells in diabetes.

## Funding

Sponsorship for this study was funded by the Takeda Korea Inc., Seoul, Republic of Korea. All authors had full access to all of the data in this study and take complete responsibility for the integrity of the data and accuracy of the data analysis.

## Declaration of competing interest

The authors declare that they have no competing interests.
