# Supplementary material for: Pioglitazone-induced AMPK-Glutaminase-1 prevents high glucose-induced pancreatic β-cell dysfunction by glutathione antioxidant system
Source: Redox Biol. 2021 Jun 3;45:102029. doi: 10.1016/j.redox.2021.102029 (PMC8187239; doi:10.1016/j.redox.2021.102029)
Supplement: Multimedia component 1 [file mmc1.pptx]

## Slide 1
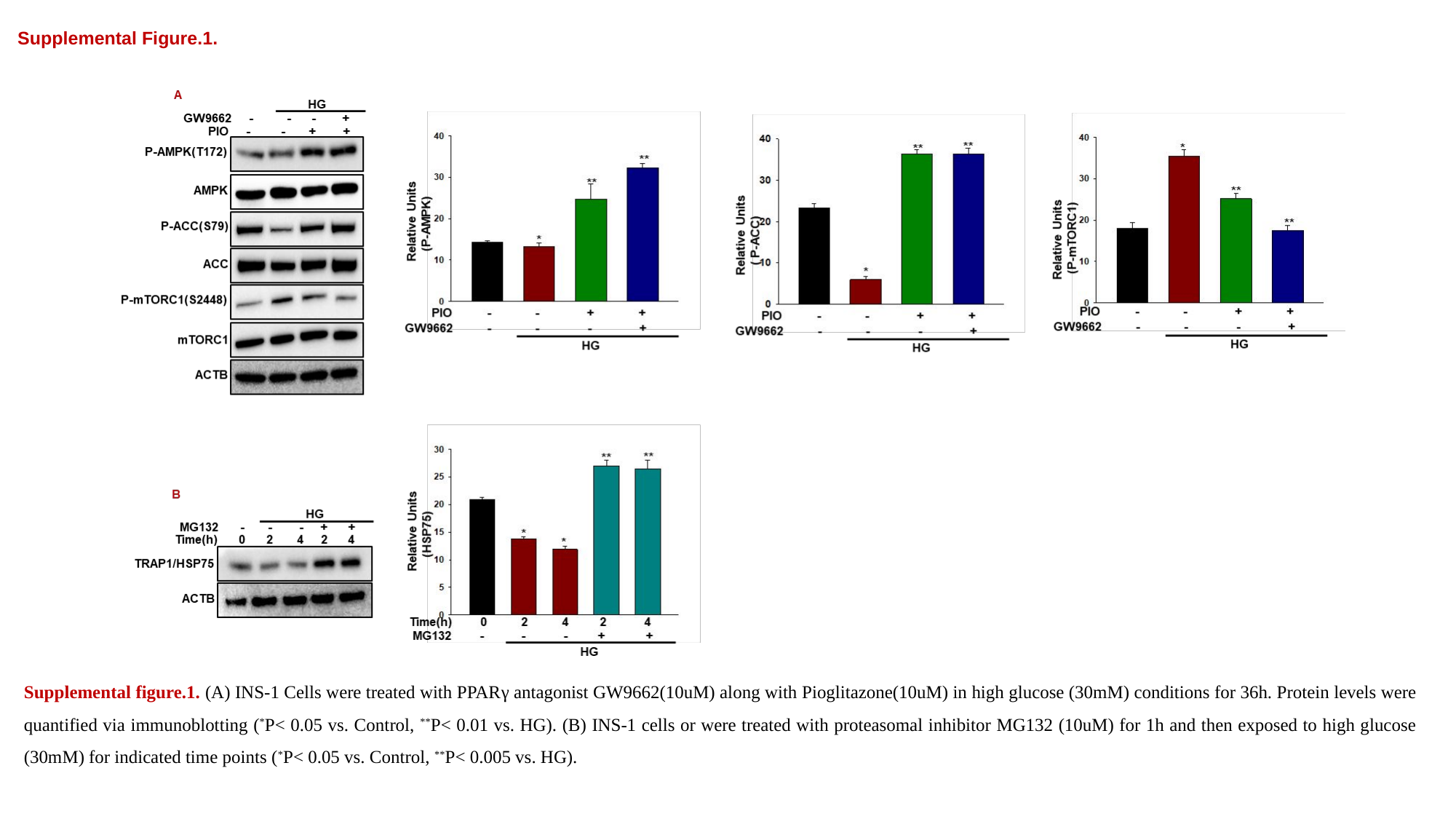

Supplemental Figure.1.
Supplemental figure.1. (A) INS-1 Cells were treated with PPARγ antagonist GW9662(10uM) along with Pioglitazone(10uM) in high glucose (30mM) conditions for 36h. Protein levels were quantified via immunoblotting (*P< 0.05 vs. Control, **P< 0.01 vs. HG). (B) INS-1 cells or were treated with proteasomal inhibitor MG132 (10uM) for 1h and then exposed to high glucose (30mM) for indicated time points (*P< 0.05 vs. Control, **P< 0.005 vs. HG).

## Slide 2
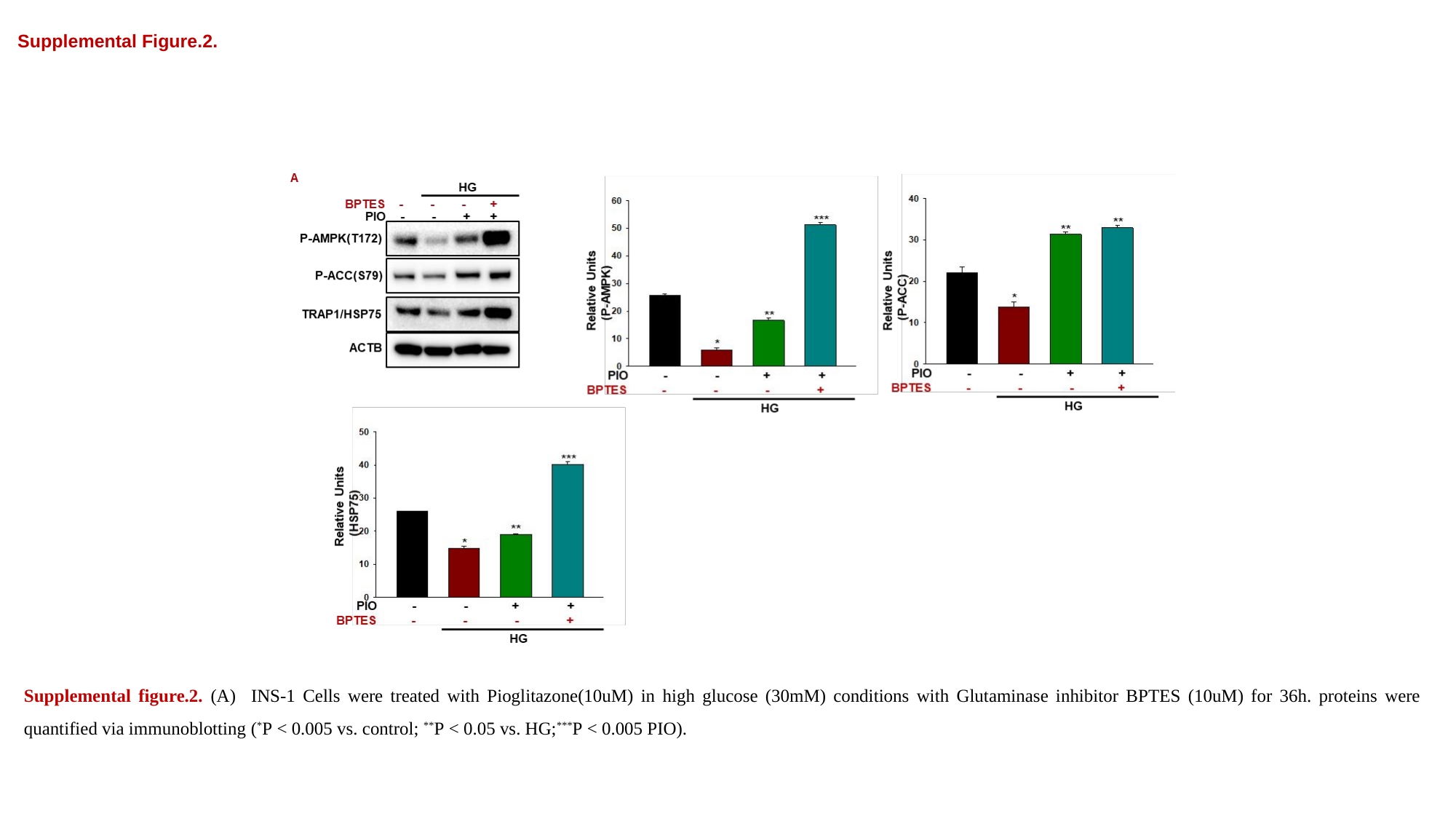

Supplemental Figure.2.
Supplemental figure.2. (A) INS-1 Cells were treated with Pioglitazone(10uM) in high glucose (30mM) conditions with Glutaminase inhibitor BPTES (10uM) for 36h. proteins were quantified via immunoblotting (*P < 0.005 vs. control; **P < 0.05 vs. HG;***P < 0.005 PIO).

## Slide 3
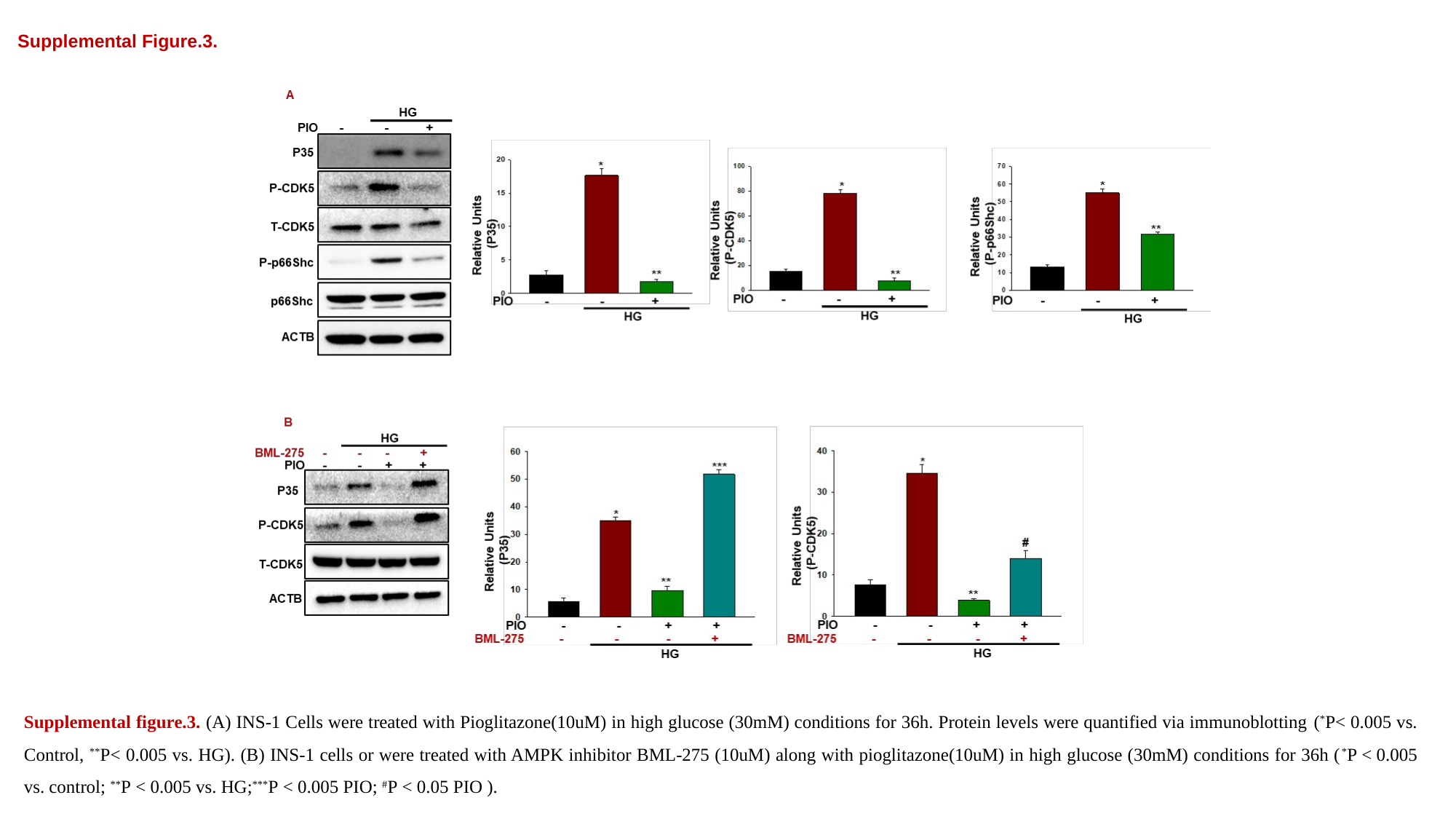

Supplemental Figure.3.
Supplemental figure.3. (A) INS-1 Cells were treated with Pioglitazone(10uM) in high glucose (30mM) conditions for 36h. Protein levels were quantified via immunoblotting (*P< 0.005 vs. Control, **P< 0.005 vs. HG). (B) INS-1 cells or were treated with AMPK inhibitor BML-275 (10uM) along with pioglitazone(10uM) in high glucose (30mM) conditions for 36h (*P < 0.005 vs. control; **P < 0.005 vs. HG;***P < 0.005 PIO; #P < 0.05 PIO ).
